# Electronically reconfigurable and conformal triband antenna for wireless communications systems and portable devices

**DOI:** 10.1371/journal.pone.0276922

**Published:** 2022-12-01

**Authors:** Musa Hussain, Esraa Musa Ali, Wahaj Abbas Awan, Niamat Hussain, Mohammad Alibakhshikenari, Bal S. Virdee, Francisco Falcone

**Affiliations:** 1 Department of Electrical Engineering, Bahria University, Islamabad Campus, Islamabad, Pakistan; 2 Faculty of Aviation Sciences, Amman Arab University, Amman, Jordan; 3 Department of Information and Communication Engineering, Chungbuk National University, Cheongju, South Korea; 4 Department of Smart Device Engineering, Sejong University, Seoul, South Korea; 5 Department of Signal Theory and Communications, Universidad Carlos III de Madrid, Leganés, Madrid, Spain; 6 Center for Communications Technology, School of Computing and Digital Media, London Metropolitan University, London, United Kingdom; 7 Department of Electric, Electronic and Communication Engineering and the Institute of Smart Cities, Public University of Navarre, Pamplona, Spain; 8 Tecnologico de Monterrey, School of Engineering and Sciences, Monterrey, Mexico; Edinburgh Napier University, UNITED KINGDOM

## Abstract

This paper presents the design of a triband antenna that can be electronically configured to operate at different frequencies. The proposed antenna is design to operate at sub-6GHz bands at 2.45 GHz (ISM, Wi-Fi, and WLAN), 3.3, 3.5 & 3.9 GHz (WiMAX), and 4.1 & 4.9 GHz (4G & 5G). This is achieved by connecting two open-ended stubs to a modified triangular patch radiator using PIN diodes. The antenna’s performance was optimized using a 3D electromagnetic solver and its performance was verified through measurements. Moreover, the conformal analysis done on the antenna shows that the proposed technique can be used in moderately flexible wireless devices without compromising the antenna’s gain, radiation efficiency and radiation patterns. These characteristics makes the proposed antenna applicable for various wireless communication systems and devices.

## 1. Introduction

5G and future 6G wireless communication systems demand small, highly compact, and multi-functional antenna systems that enable transmission of high data rates to multiple users [1]. Due to these challenges, the procedure for designing an antenna is revised. To fulfill the requirements of future communication devices, an antenna is required that has characteristics of high gain, high radiation efficiency, omnidirectional radiation pattern, and can operate in over a wideband to enable communications at multiple frequency bands [2, 3]. Conformal antennas that can be reconfigurable are considered to fulfill these demanding requirements [4].

Reconfigurable and conformal antennas have received much attention over the past few years for application in wireless communication technologies due to their many advantages. Reconfiguration in terms of frequency, radiation pattern, and polarization by using a single structure is highly desirable. A reconfigurable antenna with its flexible multi-operation feature should provide a highly compact and cost-effective solution [5]. Moreover, bendable/flexible antennas are also highly desirable as they can be mounted on curved surfaces and still allow uninterrupted and reliable wireless communications. In fact, flexible antennas are in demand for wearable devices, health monitoring systems, communication devices, and global positioning systems (GPS). The choice of the flexible antenna relies on many factors such as materials used, substrate, antenna performance, processing technique, and the surrounding environment. However, the design of flexible antennas must address challenges of resonant frequency shift and impedance mismatch due to the variation of effective capacitance during bending of the antenna. The challenge is the design of an antenna that combines the characteristics of reconfigurability and flexibility [6, 7].

Several antenna designs reported in literature have characteristics of wideband, high gain, and reconfigurability. However, most of these antennas are implemented on rigid materials. In [8] the antenna reported has dimensions of 30×27×1.6 mm^3^ and operates over a wideband covering ISM and S-band; however, the antenna is only suitable for rigid devices. In [9], the dual-band antenna operating at 4.5 GHz and 6.5 GHz is for S-band applications. The frequency of the antenna’s characteristics can be reconfigured however the antenna has a narrow band and it’s designed on a rigid material. The antenna reported in [10] has an electrically small size of 22×22 mm^2^ and operates at 3.5 GHz and 5.5 GHz. This antenna too has a narrow bandwidth of 0.7 GHz and 0.6 GHz at the two respective resonant frequencies.

Although antennas reported in [11, 12] are compact and have a high gain >4 dBi over a wideband they however have structural complexity and fabricated on a rigidFR-4 material. In [13], the dual-band antenna is flexible and reconfigurable. It and operates at 1.88 GHz and 2.1 GHz however it has a narrow bandwidth of 0.024 GHz and 0.015 GHz, respectively. The quad-band reconfigurable antenna in [14] operates at S-band (4.5 GHz, 4.8 GHz, 5.2 GHz, and 5.8 GHz) and has a peak gain of 4 dBi however it too has narrow bandwidths of 0.2 GHz, 0.7 GHz, 0.02 GHz, and 0.6 GHz, respectively.

The high gain antenna in [15] has a gain of 5.5 dBi and 5 dBi atits resonance frequencies of 2.45 GHz and 3.5 GHz, respectively. The antenna can be switched to operate at either of its resonance frequencies using PIN diodes. The drawback of this antenna is its large size (100×100×2.5 mm^3^) and structural complexity due to the differential excitation technique employed. In [16] a compact dual-band antenna is reported of size 27×6×1.6 mm^2^that operates in the ISM and WLAN bands. Although this antenna has simple geometry and peak gain of 3 dBi however it has a narrow bandwidth of 0.3 GHz and 0.2 GHz at 2.4 GHz and 5.2 GHz, respectively. In [17], the reconfigurable frequency antenna can be tuned between 2.5 GHz and 3.5 GHz. A via-port for biasing the PIN diodes is implemented on the truncated ground plane.

The reconfigurable dual-band antenna reported in [18] for WLAN applications has a peak gain of 5.6 dBi at 5 GHz and 5.5 GHz with bandwidths of 0.2 GHz and 0.15 GHz, respectively. The antenna has a complex geometry and its footprint size of 40×40×0.762 mm^3^ limit is usage for other wireless applications. In [19] a high gain antenna is reported for 5G and Wi-Fi/Bluetooth applications. The antenna has a gain of 7.68 dBi at 2.8 GHz and 4.8 GHz with corresponding bandwidth of 1 GHz and 0.75 GHz, respectively. Although the geometry of the antenna is simple its design is for a rigid substrate of size 40×26×1.6 mm^3^. A frequency reconfigurable and flexible dual-band antenna for 5G applications reported in [20] operates at 3.5 GHz and 5.5 GHz with a peak gain of 3.2 dBi. Its disadvantages are a large size and complex geometry.

In [21], the tri-band reconfigurable antenna operates at the ISM, WiMAX, WLAN and 5G bands. However, it has a narrow bandwidth of 0.2 GHz, 0.3 GHz, and 0.3 GHz at its resonance frequencies of 2.45 GHz, 3.5 GHz, and 5.2 GHz, respectively. It also has a relatively large size of 53×35×1.6 mm^3^. Reported in [22] is a co-planner waveguide fed, dual-band antenna of size 42×36×2.4 mm^3^ operates at 2.25 GHz and 3.2 GHz for WLAN and WiMAX applications. Here via-ports placed at various layers are used to achieve reconfiguration, which leads to a complex structure not easily fabricable. An antenna based on a circular shaped patch is shown to offer dual-band characteristics applicable for ISM and WLAN systems in [23]. Although the antenna has simple geometry however it has a narrow bandwidth of 0.75 GHz at its resonance frequency of 2.45 GHz and low gain of 0.9 dBi.

An UWB antenna reported in [24] has a large bandwidth of 2–22.9 GHz and a high gain. Although it has relatively small dimensions of 27×17×1.6 mm^3^the antenna design is complex. In [25] a complex multi-band antenna design is presented for ISM, WLAN, and WiMAX applications. It has of dimensions 40×32×1.6 mm^3^ but a low gain of 1.8 dBi. On-demand frequency reconfigurable antenna reported in [26] offers a wideband in a compact size of 21×15 mm^2^. This antenna can be reconfigured in eight bands by using two PINdiodes, but the antenna suffers from narrow bands and low gain. Another reconfigurable antenna which is based on T-shaped structure reported in [27] can be switched using PIN diodes to operate over a tribandat 2.4 GHz, 3, and 5.2 GHz. This antenna too suffers from narrow bands.

It can be discerned from the above overview reported in the literature are numerous reconfigurable antennas applications in various bands including ISM, WLAN, WiMAX and 5G. Although many of these antennas have desirable features of compact size and simple design geometry however their design is limited to rigid structure and in most cases, they have a relative low gain and narrow band performance. Moreover, although some designs may offer higher gains however their drawback is complex structural designs which can be costly to manufacture. The above study shows there is scope to design a wideband reconfigurable antenna on a flexible substrate for multi-band applications that is based on a simple geometry of a compact size and offers high gain. In this paper, such an antenna is proposed that is fabricated on a flexible substrate and can be reconfigured using PIN diodes for application over a wideband covering sub-6GHz systems including ISM, WiMAX, 4G and 5G.

The rest of the paper is divided into three sections. Section II discusses the antenna design methodology, proposed antenna geometry, and working principle. In section III, discussed are the hardware prototype, measured results along with comparison of the proposed antenna with the state-of-the-artwork. The paper is concluded in section IV.

## 2. Design methodology of proposed wideband triband antenna

### 2.1 Antenna geometry

**Fig 1:**
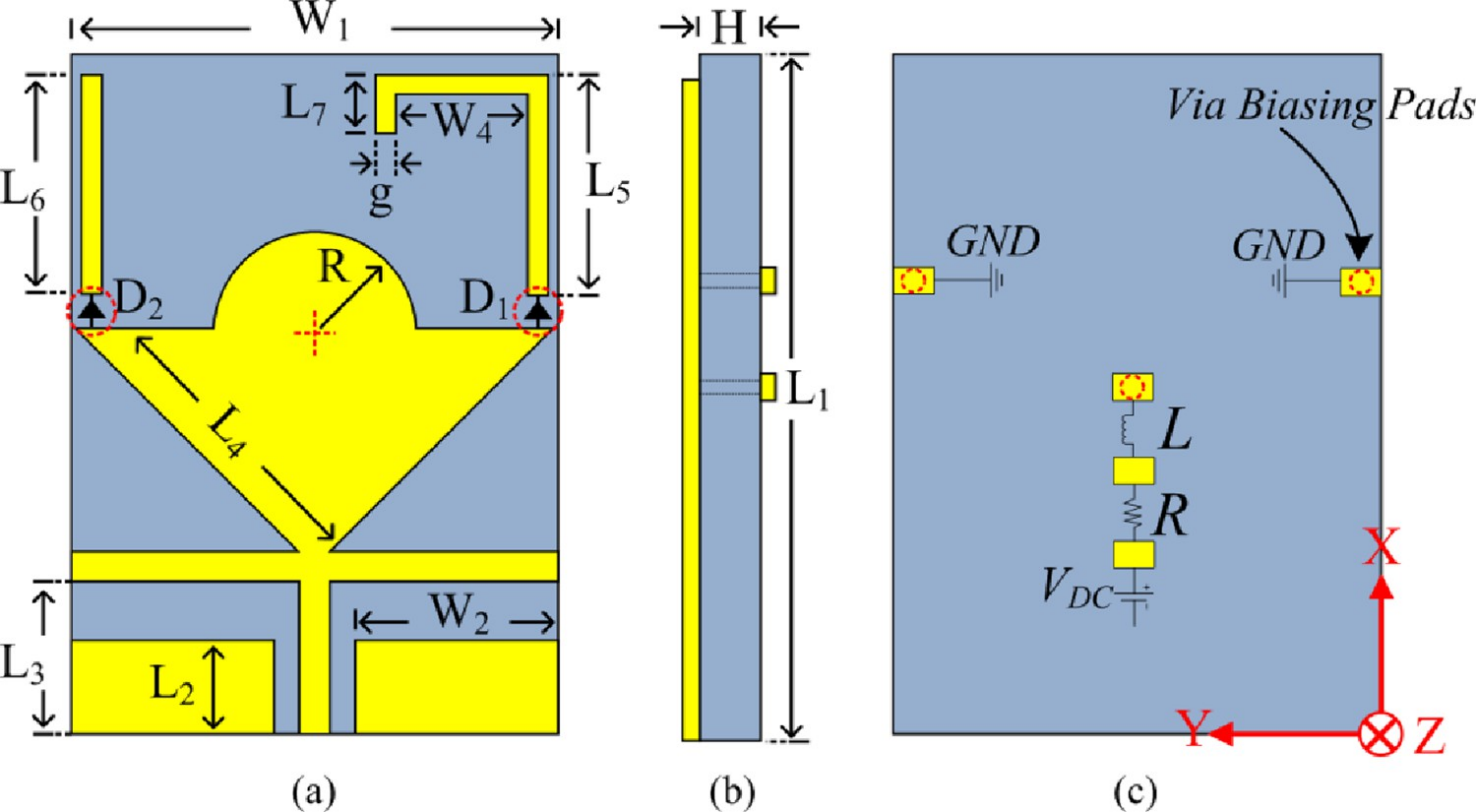
The geometry of proposed CPWfed frequency reconfigurable antenna operating over a wideband including ISM, WLAN and 5G wireless technologies, (a) Front view, (b) Side view, and (c) back view.

The geometry of the proposed wideband antenna for triband applications including 5G systems and devices is shown in Fig 1. The antenna consists of an edge-fed inverted triangular patch radiator with a semi-circular protrusion, as shown in Fig 1. The antenna is excited via a co-planar waveguide (CPW) feedline. The advantage of CPW fed slot antenna is its wide band characteristics. The upper two corners of the triangular-shaped radiator are connected to two open-circuited microstrip stubs of different lengths via PIN diodes D1 and D2. Equal length open-circuit stubs oriented horizontally are located at the junction of the feedline and the radiator to improve the impedance match of the antenna for S_11_≤-10 dB. The antenna is matched to 50Ω feedline by adjusting the gap between the feedline and the ground plane. DC biasing of the PIN diodes is implemented from the backside of the substrate board. The antenna is etched on the top side of a flexible substrate, i.e, Rogers RT/duroid 5880 of relative permittivity = 2.2, thickness of 0.254 mm, and loss tangent of 0.0009. The antenna’s performance was optimized using the 3D electromagnetic solver High-Frequency Structural Simulator (HFSS). The optimized parameters of proposed antenna are: *L*_*1*_ *= 30*mm, *W*_*1*_ *= 25*mm, *L*_*2*_ *= 5*mm, *L*_*3*_ *= 6*mm, *L*_*4*_ *= 14*mm, *L*_*5*_ *= 12*mm, *L*_*6*_ *= 12*mm, *L*_*7*_ *= 6*mm, *W*_*2*_ *= 11*mm, *W*_*4*_ *= 8*mm, *R = 6*mm, *g = 1*mm, and *H = 0*.*254*mm. The overall antenna dimensions are 30×25×0.254 mm^3^.

### 2.2 Design steps

**Fig 2:**
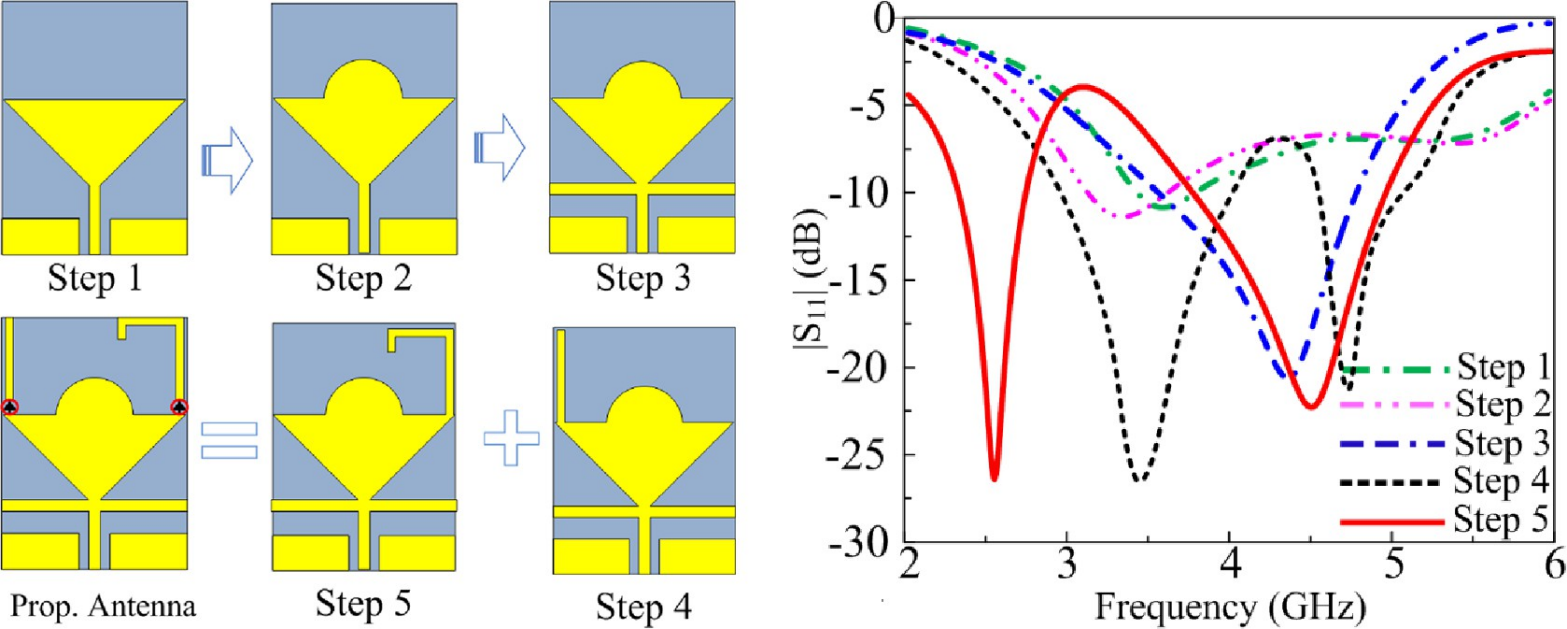
(a) Design evolution of proposed antenna, and (b) impact of the various steps on antenna’s |S_11_|response.

The steps taken to realize the final proposed CPW fed triangular-shaped monopole antenna are shown in Fig 2(A). The triangular-shaped monopole antenna was designed at a center frequency of 3.5 GHz using equations given in [28]. The resulting reflection-coefficient response of the antenna in Fig 2(B) shows the impedance bandwidth for S_11_≤-10 dB of the standard inverted triangular-shaped antenna is limited. In the second step a semi-circular patch is added at the top of the radiator and the corresponding reflection-coefficient response shows improvement in the impedance bandwidth. In the third step open-circuited stubs of equal length are attached to the feedline at the interface with the patch antenna. As evident in Fig 2(B) this step significantly improves the reflection-coefficient response but degrades the bandwidth. In the fourth step a vertical high impedance open-circuited stub is attached to the left-hand corner of the radiator. The action of this results in introducing two resonance modes at 3.5 GHz and 4.8 GHz with bandwidths ranging from 2.9–4 GHz and 4.6–5 GHz, respectively, and impedance match better than -25 dB. In the fifth step another vertical stub is added to the right-hand corner of the radiator. This stub maintains the dual-band resonance but they bands are shifted lower in frequency at 2.5 GHz and 4.5 GHz, as shown in Fig 2(B), with corresponding bandwidths ranging from 2.3–2.7 and 3.9–5 GHz, respectively. The proposed antenna is based on step five, but the vertical stubs are connected to the radiator through PIN diode switches to realize a reconfigurable architecture. The antenna offers triband capability over a wideband by switching the appropriately switching the PIN diodes ‘on’ or ‘off’.

### 2.3 Working principle

The proposed antenna consists of two PIN diodes D_1_ and D_2_, which connect the triangular-shaped radiator to the left-hand side and right open-circuited stubs with corner edges of the triangular radiator. When only D_1_ is configured in forward DC bias mode, the right-hand stub along with a triangular patch antenna radiate. When only D_2_ is forward biased, then left-hand side stub and the triangular patch antenna radiate. The two PIN diodes enable various biasing scenarios as described below.

#### Case 00

In this case, the two diodes are reverse biased and there is no connection between the two stubs and radiating patch. Hence, the patch antenna alone radiates at 4.4 GHz.

#### Case 01

In this scenario, the one PIN diode D_2_is forward biased, i.e., switched ‘on’, and thus connected to the patch antenna. PIN diode D_1_ is reverse biased (switched ‘off’) and not connected to the patch antenna. The total length of L_6_ is approximately equal to λ/4. Hence, this antenna configuration radiates at 3.5 GHz and 4.8 GHz.

#### Case 10

In the scenario, PIN diode D_1_is forward biased and D_2_ is reverse biased. Hence, the right-hand stub is connected to the triangular radiator. The antenna under this condition radiates at 2.5 GHz and 4.5 GHz.

#### Case 11

In the final scenario, both PIN diodes are switched ‘on’. The left and right-hand side stubs are connected to the patch antenna. In addition to the resonance modes generated when the diodes are separately switched ‘on’ the antenna resonates at an additional frequency of 2.45 GHz.

## 3. Simulated and measured results

**Fig 3:**
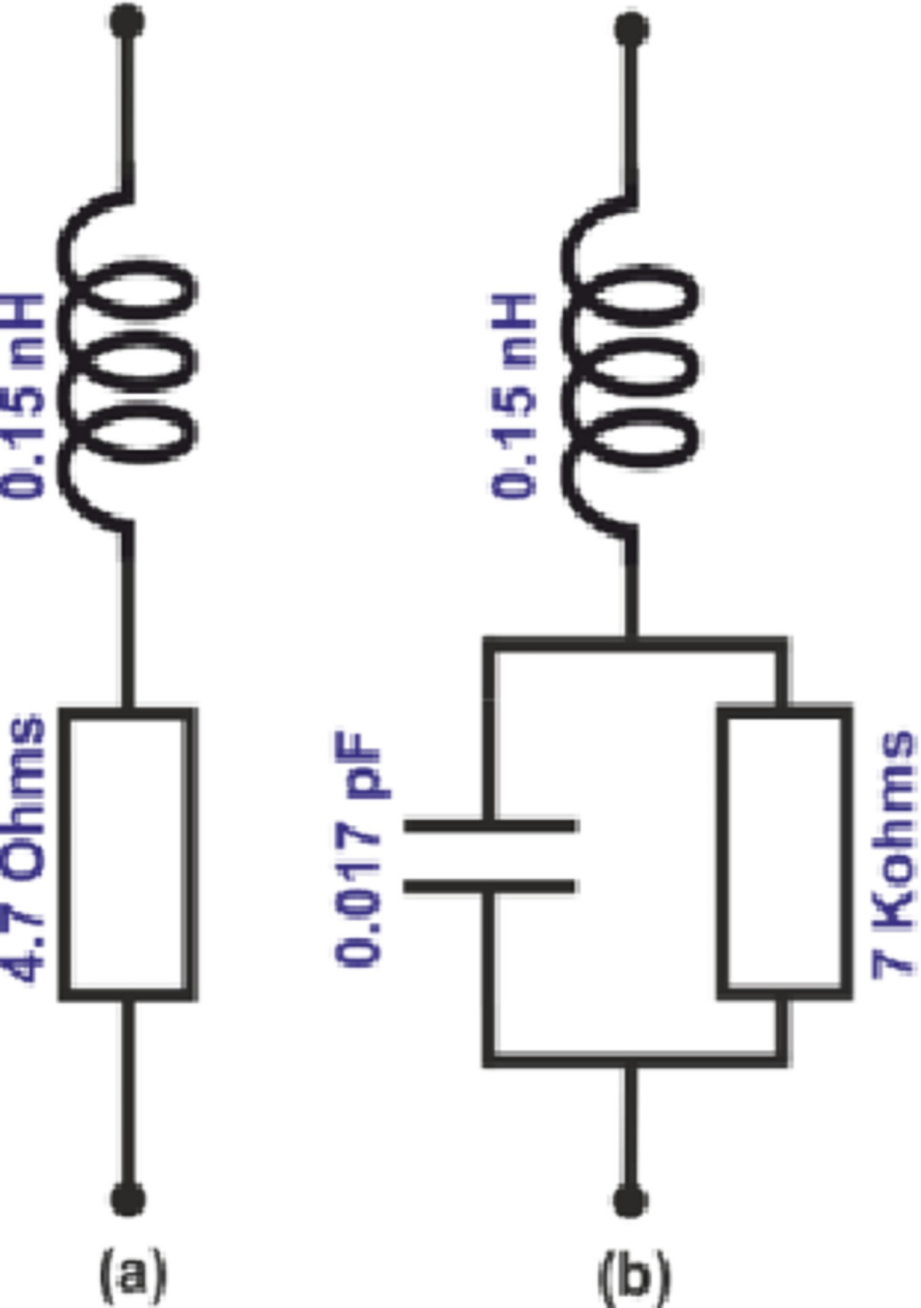
Equivalent circuit of diode for the PIN diode, (a) Forward biased, and (b) Reverse biased.

**Fig 4:**
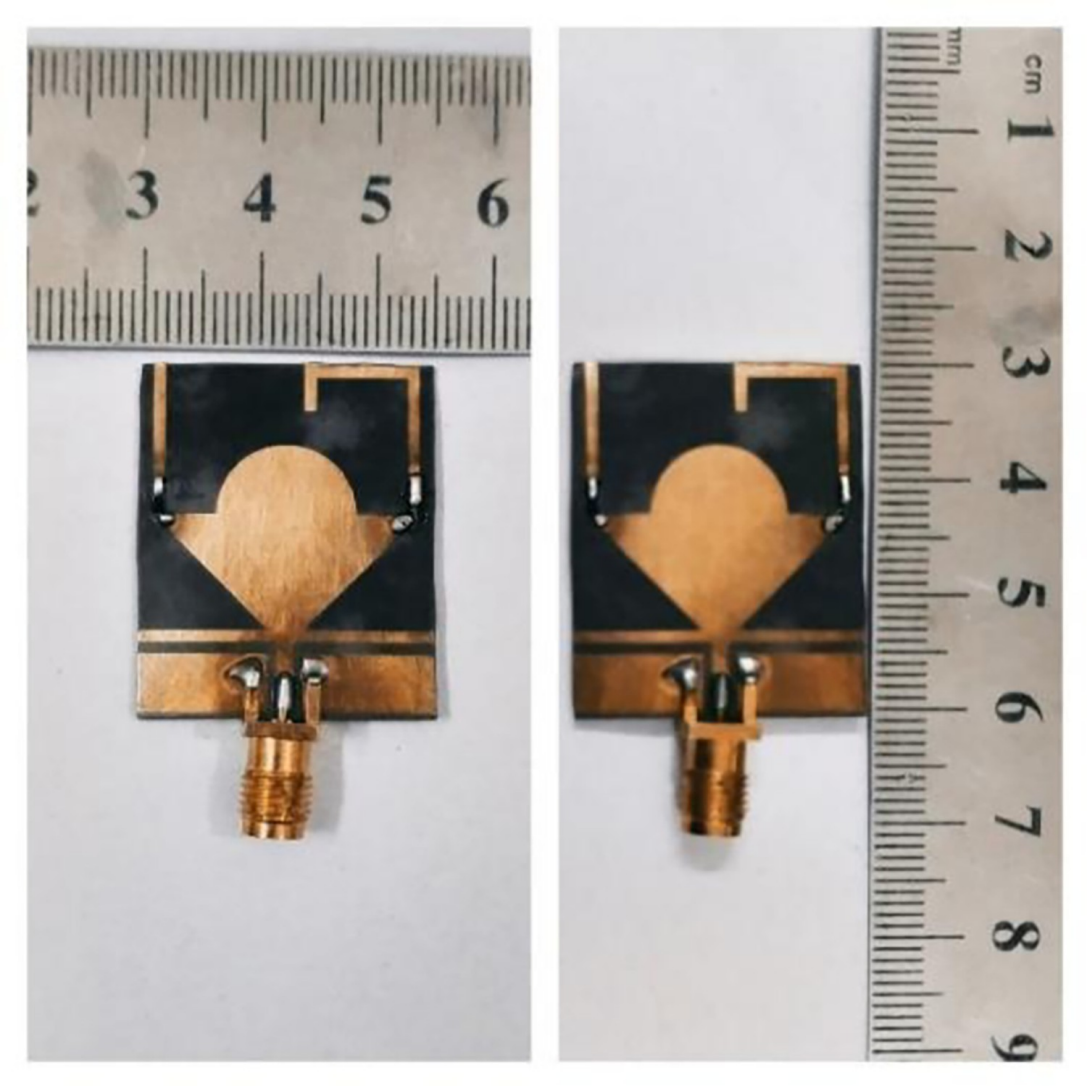
Prototype of proposed frequency reconfigurable and flexible antenna.

This section presents the simulated and measured results of the proposed reconfigurable antenna. Fig 3 shows the equivalent circuit model of the PIN diode used in the proposed antenna in the forward (switch ‘on’) and reverse bias (switch ‘off’) conditions [29]. The fabricated antenna is shown in Fig 4. The antenna’s reflection-coefficient and gain performance were measured using a Vector Network Analyzer (VNA) model HP 8720D (50 MHz– 13.5 GHz). The radiation patterns were measured in an anechoic chamber. The receive antenna used was a standard broadband horn antenna (1–18 GHz) EMCO Type 3115. The gap between the proposed test antenna and the reference horn antenna was 3m.

### 3.1 Case-00

**Fig 5:**
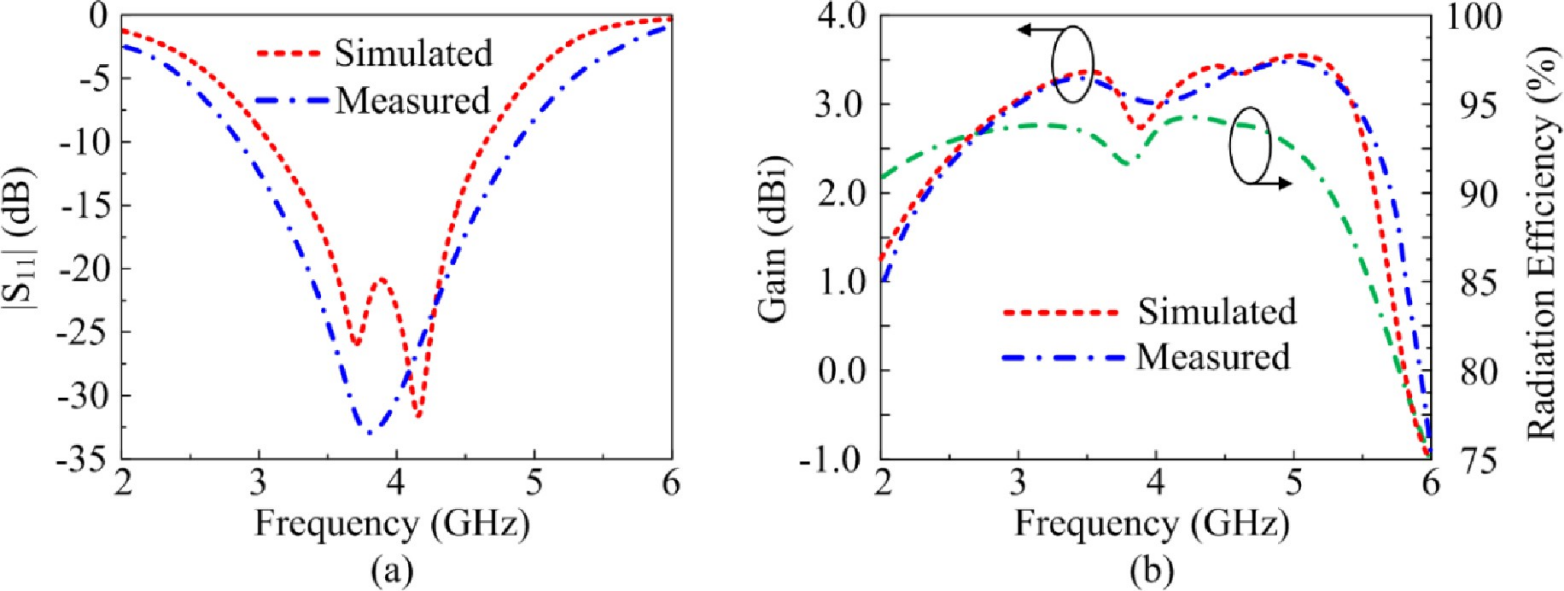
The reflection-coefficient, gain and radiation efficiency response of the proposed antenna when both PIN diodes are reverse biased.

**Fig 6:**
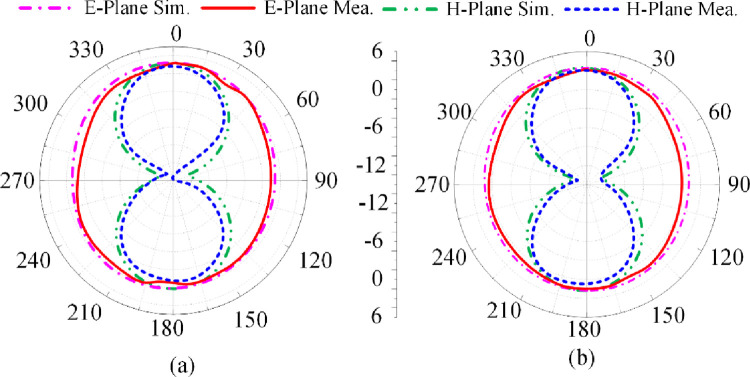
Radiation pattern of proposed antenna when both PIN diodes are reverse biased at (a) 3.7 GHz, and (b) 4.2 GHz.

Fig 5 shows the proposed antenna’s reflection-coefficient, gain and radiation efficiency when both PIN diodes are switched ‘off’ by reverse biasing them. Under this biasing condition only the triangular-shaped patch antenna with the horizontal open-circuited stubs radiate. The antenna resonates at 3.85 GHz having a measured impedance bandwidth of 2.1 GHz from 2.8–4.9 GHzfor S_11_≤ -10 dB with a peak reflection-coefficient dip at 3.75 GHz. The measured gain is >2 dBi between 2.25 GHz and 5.75 GHz with a peak gain of 3.5 dBi at 5.1 GHz. The measured radiation efficiency between 2.25 GHz and 5.75 GHz is >90% with a peak efficiency of 94.5% at 4.2 GHz. The measured radiation pattern at the resonance frequencies of 3.7 GHz and 4.2 GHz are given in Fig 6. In the E-plane the antenna radiates energy omnidirectionally however in the H-plane it radiates bidirectionally. The radiation patterns at both frequencies are similar. There is good agreement between the measured and simulation results.

### 3.2. Case-01

**Fig 7:**
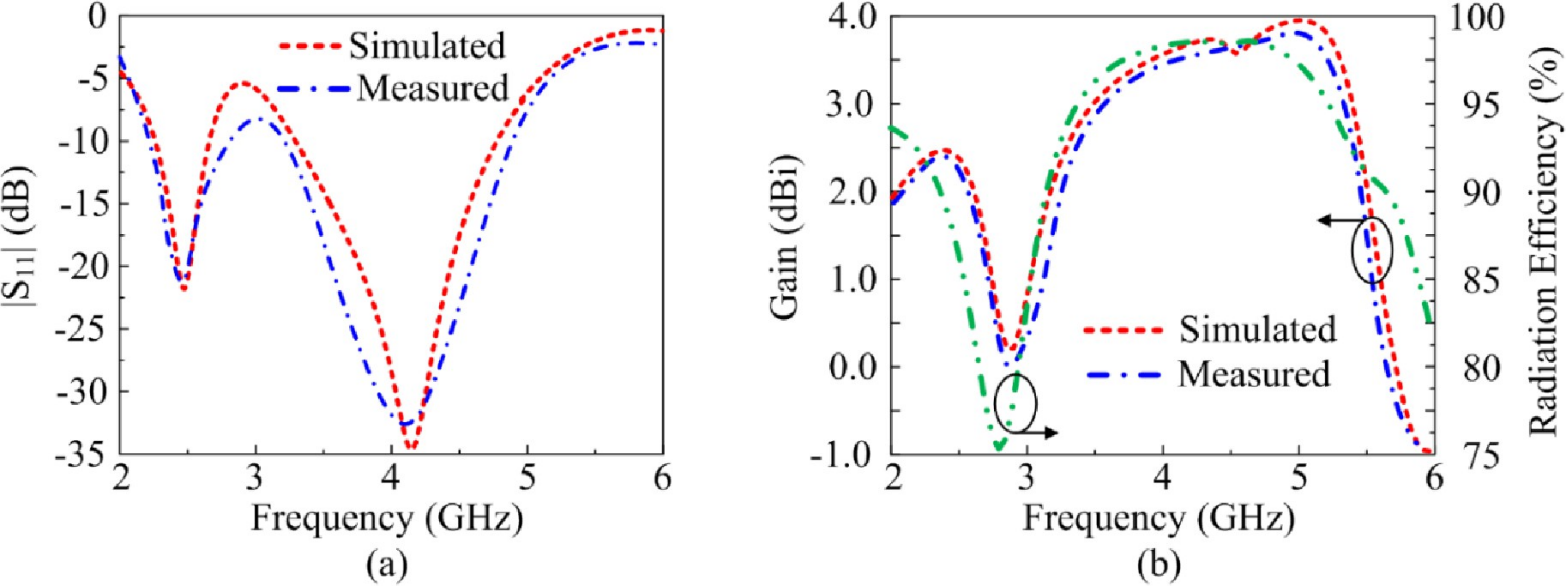
The reflection-coefficient, gain and radiation efficiency response of the proposed antenna when PIN diode D1 is reverse biased and D2 is forward biased.

**Fig 8:**
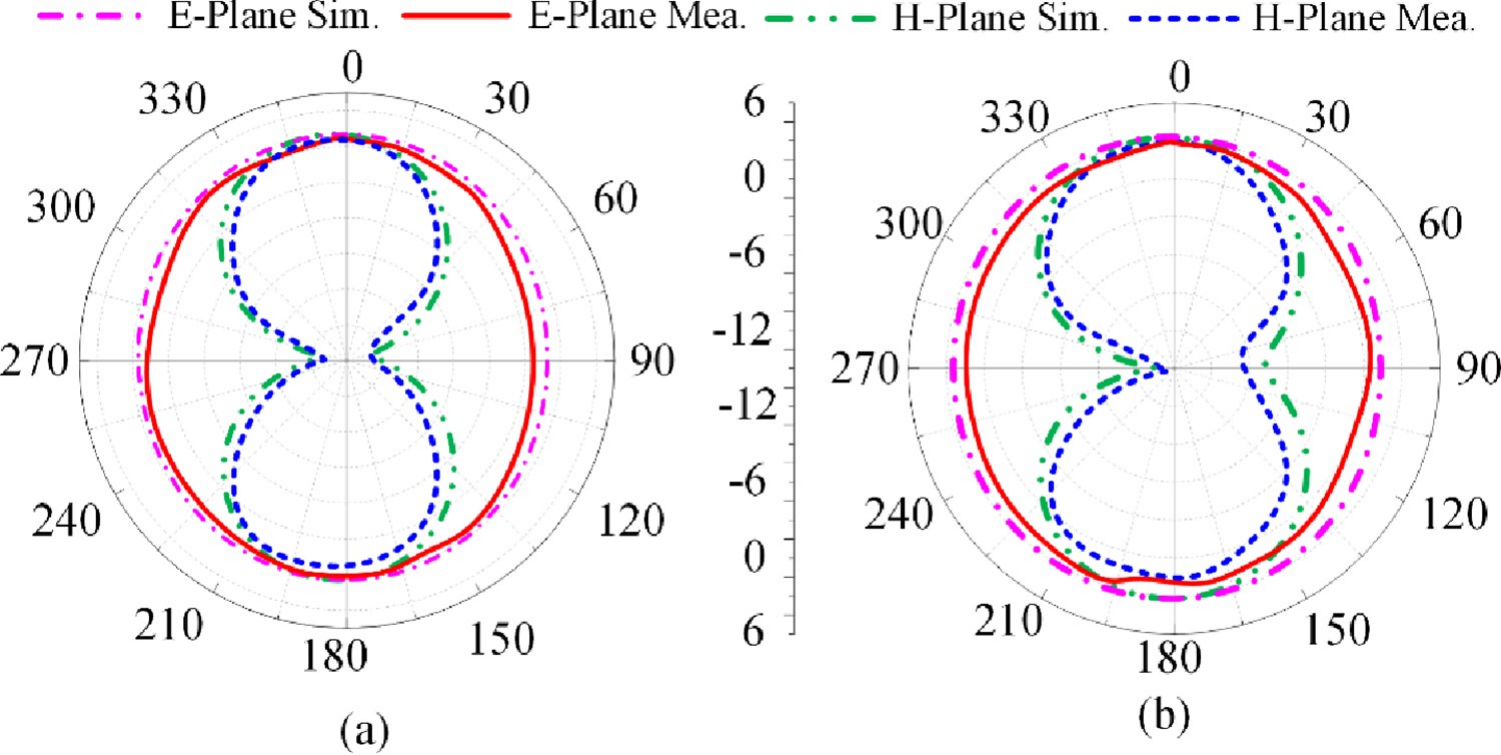
Radiation pattern of proposed antenna when PIN diode D1 is reverse biased and D2 is forward biased at (a) 2.4 GHz, and (b) 4.1 GHz.

The proposed antenna’s reflection-coefficient, gain and radiation efficiency when PIN diode D1 is switched ‘off’ and 2 is switched ‘on’ are shown in Fig 7. In this scenario the left-hand vertical stub is connected to the radiating patch. The consequence of this is excitation of dual-band at 2.5 GHz and 4.1 GHz. The bandwidth at 2.5 GHz for S_11_≤ -10 dB is 0.5 GHz from 2.25–2.75 GHz, and at 4.1 GHz bandwidth is 1.62 GHz from 3.2–4.82 GHz. The measured gain is >2 dBi between 2 GHz and 2.6 GHz, and between 3.25 GHz and 5.5 GHz. The peak gain is 3.9 dBi at 5.1 GHz. The measured radiation efficiency between 2–2.6 GHz and 3.25–5.5 GHz is >89.5% with a peak efficiency of 99% at 5.1 GHz. The measured radiation pattern at the resonance frequencies of 2.5 GHz and 4.1 GHz are given in Fig 8. In the E-plane the antenna radiates energy omnidirectionally however in the H-plane it radiates bidirectionally. The radiation patterns in the E-plane at both frequencies are similar however the radiation pattern in the H-plane at 4.1 GHz is distorted. There is good agreement between the measured and simulation results.

### 3.3 Case-10

**Fig 9:**
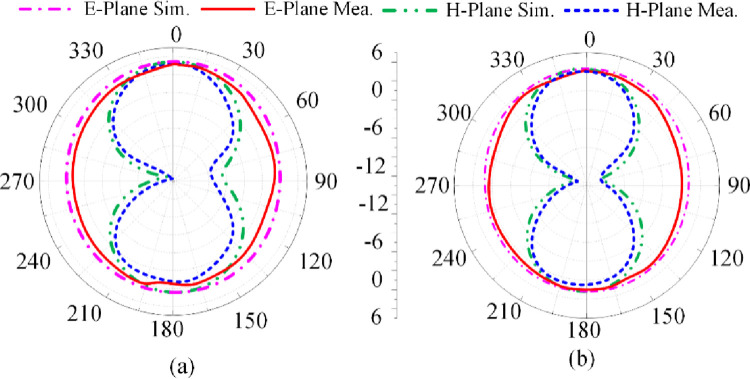
Radiation pattern of proposed antenna when PIN diode D1 is forward biased and D2 is reverse biased at (a) 3.5 GHz, and (b) 4.8 GHz.

**Fig 10:**
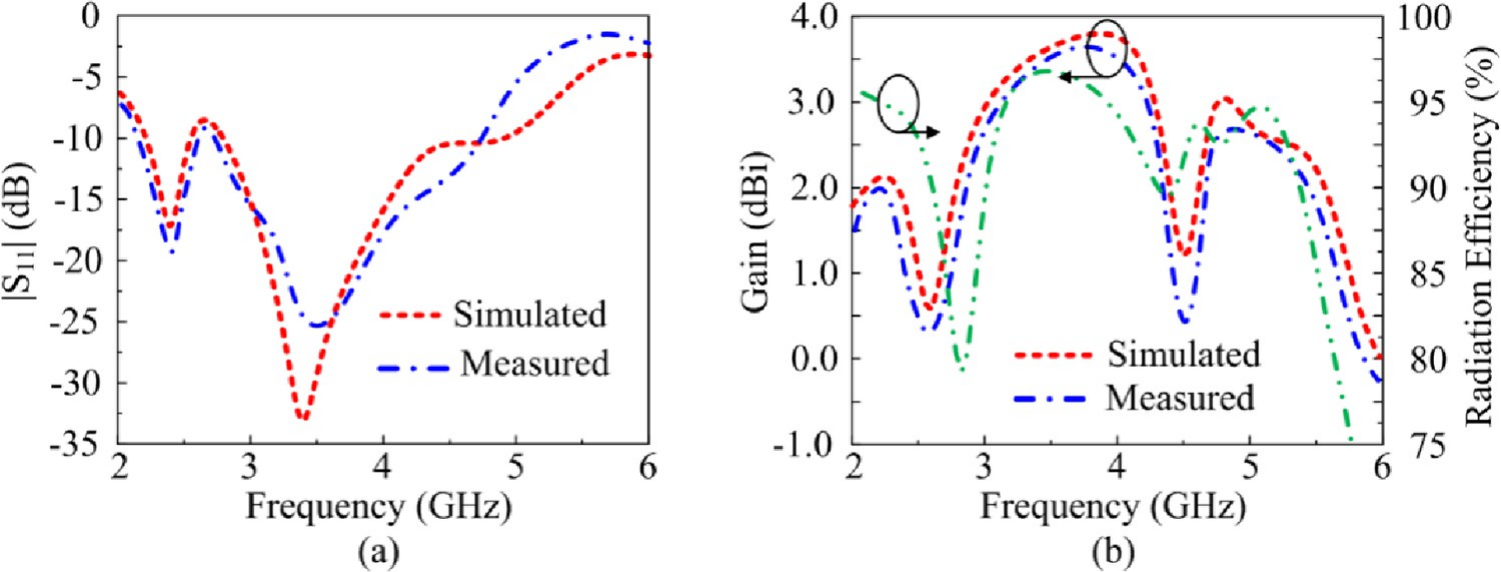
The reflection-coefficient, gain and radiation efficiency response of the proposed antenna when both PIN diodes are forward biased.

Fig 9 shows the proposed antenna’s reflection-coefficient, gain and radiation efficiency when PIN diode D1 is switched ‘on’ and 2 is switched ‘off’. In this scenario the right-hand vertical stub is connected to the radiating patch. Once again dual-band is excited at 3.5 GHz and 4.8 GHz. The bandwidth at 3.5 GHz for S_11_≤ -10 dB is 1.25 GHz from 2.75–4 GHz, and at 4.8 GHz bandwidth is 1 GHz from 4.4–5.4 GHz. The measured gain is >2 dBi between 2.1 GHz and 4 GHz, and between 4.5 GHz and 5.6 GHz. The peak gain is 3.5 dBi at 5 GHz. The measured radiation efficiency between 2.75–4 GHz and 4.4–5.4 GHz is >90% with a peak efficiency of 98.5% at 3.4 GHz. The measured radiation pattern at the resonance frequencies of 3.5 GHz and 4.8 GHz are given in Fig 10. In the E-plane the antenna radiates energy omnidirectionally however in the H-plane it radiates bidirectionally. The radiation patterns in the E-plane at both frequencies are similar however the radiation pattern in the H-plane at 3.5 GHz is distorted. There is good agreement between the measured and simulation results.

### 3.4. Case-11

**Fig 11:**
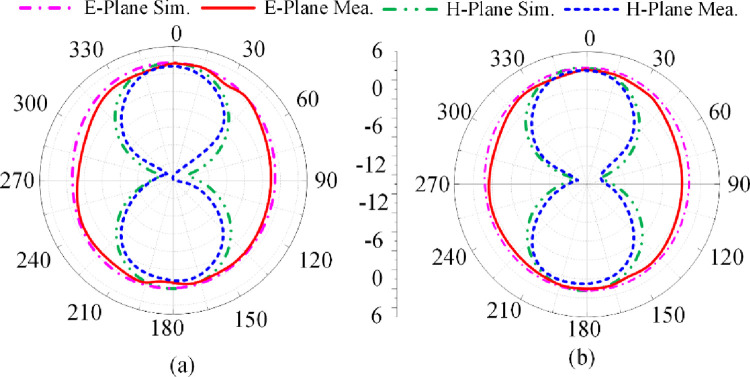
Radiation pattern of proposed antenna when both PIN diodes are forward biased at (a) 2.45 GHz, and (b) 3.5 GHz.

The reflection-coefficient, gain and radiation efficiency of the proposed antenna when both PIN diodes are switched ‘on’ are shown in Fig 10. In this case both the vertical stubs are connected to the radiating patch. The reflection-coefficient shows the antenna to resonate at 2.45 GHz and at 3.5 GHz. The resonance at 3.5 GHz is much more pronounced than at 2.45 GHz. The bandwidth at 2.45 GHz for S_11_≤ -10 dB is 0.55 GHz from 2.1–2.65 GHz, and at 3.5 GHz bandwidth is 2.15 GHz from 2.65–4.8 GHz. The measured gain is >2 dBi between 2.85 GHz and 4.4 GHz, and between 4.65 GHz and 5.5 GHz. The peak gain is 3.75 dBi at 3.9 GHz. The measured radiation efficiency between 2.85–4.4 GHz and 4.65–5.5 GHz is >90% with a peak efficiency of 98.5% at 3.8 GHz. The measured radiation pattern at the resonance frequencies of 2.45 GHz and 3.5 GHz are given in Fig 11. In the E-plane the antenna radiates energy omnidirectionally however in the H-plane it radiates bidirectionally. The radiation patterns in the E-plane at both frequencies are similar however the radiation pattern in the H-plane at 3.5 GHz is slightly wider at center. There is good agreement between the measured and simulation results.

### 3.5 Conformal analysis

**Fig 12:**
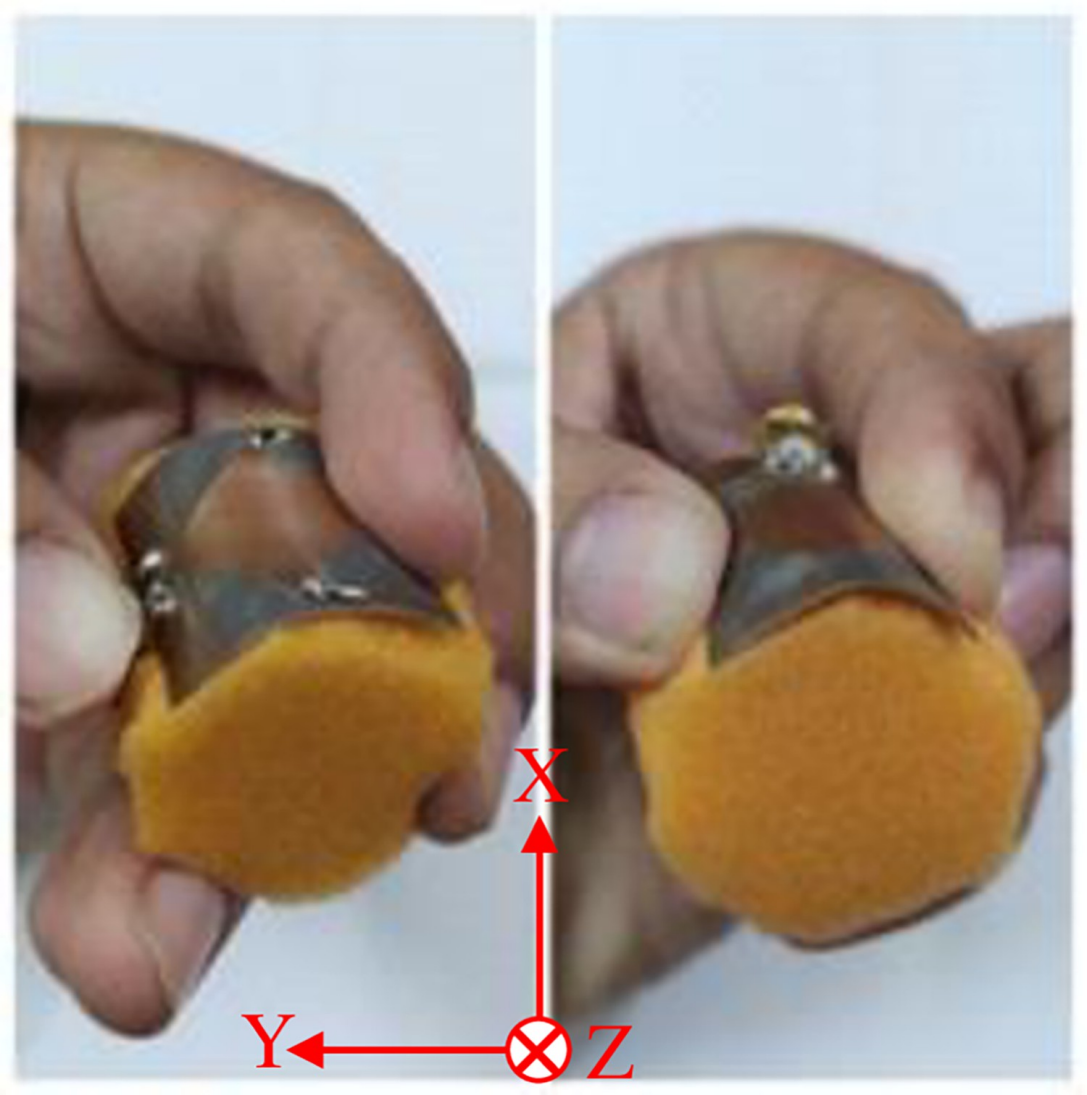
The prototype frequency reconfigurable antenna under the conformal condition.

**Fig 13:**
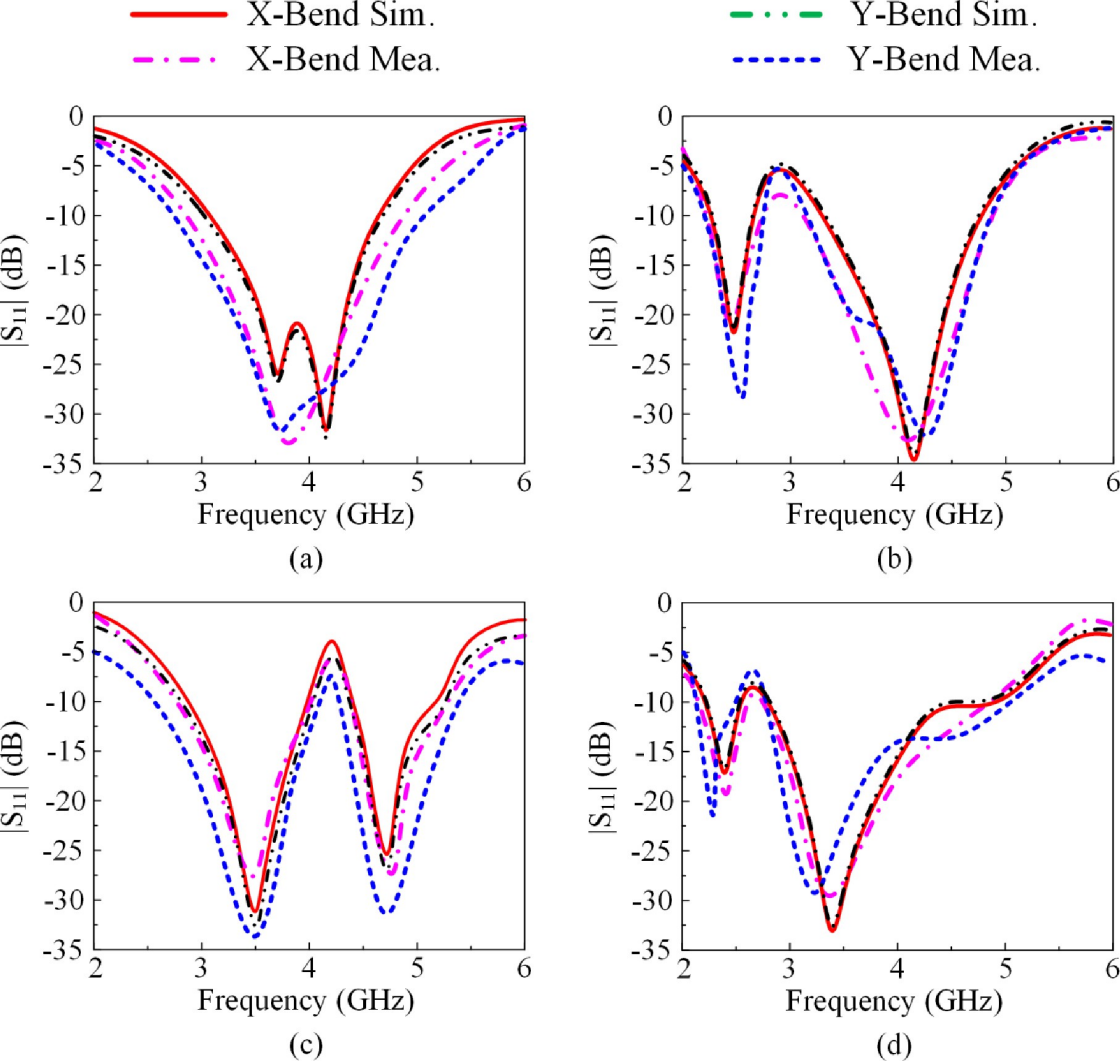
The reflection-coefficient response of the proposed antenna when wrapped around a cylinder under various PIN diode switching scenarios, (a) case-00, (b) case-01, (c) case-10, and (d) case-11.

**Fig 14:**
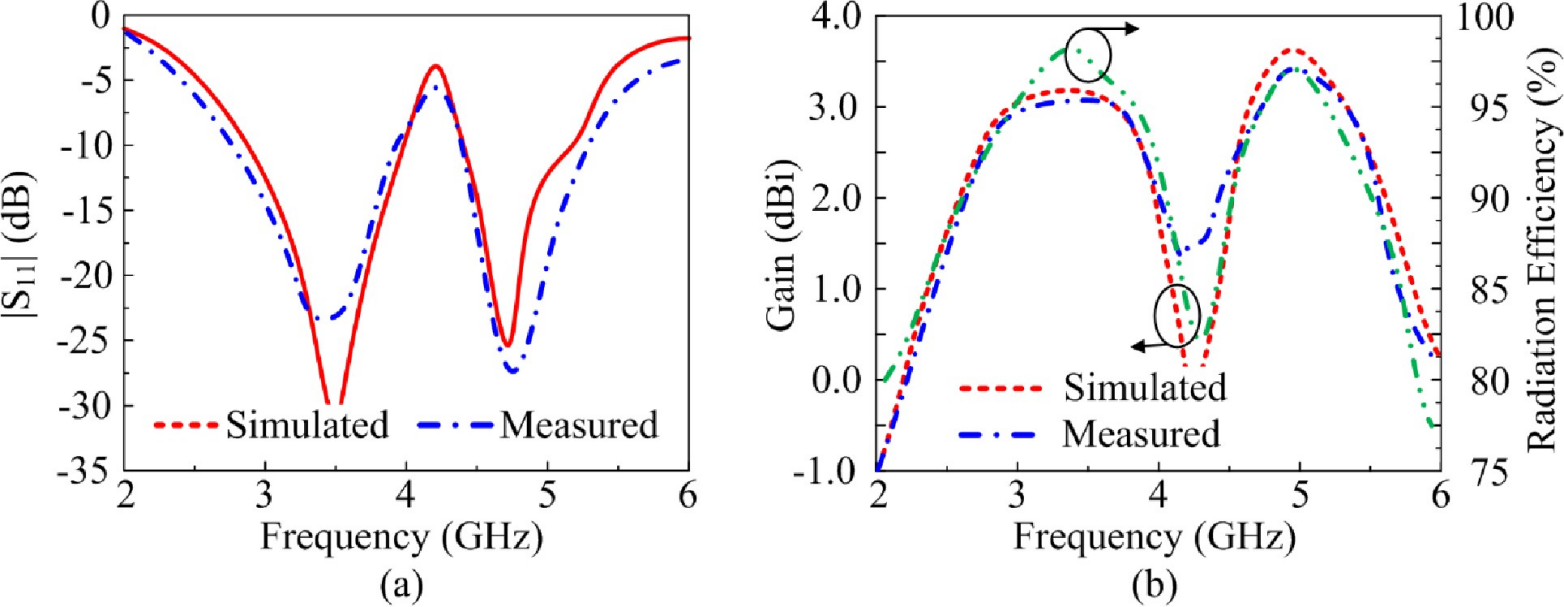
The reflection-coefficient, gain and radiation efficiency response of the proposed antenna when PIN diode D1 is forward biased and D2 is reverse biased.

This section shows how the performance of the proposed antenna is affected under bending conditions shown in Fig 12. The antenna was bent along the *xy*-plane by wrapping it on cylinder having radius of 25 mm. Fig 13 shows reflection-coefficient response of the proposed antenna when wrapped around a cylinder under various PIN diode switching scenarios. Comparison of the reflection-coefficient response with no bending in Figs 5, 7, 10 and 14 shows there is no effect on the reflection-coefficient response. The demonstrates that the proposed antenna can be used in both non-conformal and conformal scenarios.

The performance of the proposed antenna under the various PIN diode switching conditions are given in Table 1. It can evident that the antenna maintains the peak gain above 3.5 dBi and the radiation efficiency above 94% under the different switching modes. The table shows the proposed antenna can be configured electronically to operate at various bands including ISM, WLAN, 4G and 5G.

**Table 1:** Antenna performance under various PIN diode switching conditions.

Case	Mode of Operational	Measured Band (GHz)	Peak measured gain (dBi)	Peak efficiency (%)
**00**	Wideband	2.8–4.9	3.5	94.5
**01**	Dual-band	2.25–2.75	3.9	99
		3.2–4.82		
**10**	Dual-band	2.75–4	3.5	98.5
		4.4–5.4		
**11**	Dual-band	2.1–2.65	3.75	98.5
		2.65–4.8		

Table 2 represents the comparison of the salient features of the proposed antenna with other state-of-the-art dual-band and triband antennas reported in the literature. Compared to the triband antenna of [21] the proposed triband antenna has a smaller footprint size, higher gain and can be implemented on a flexible substrate. The only other flexible antenna cited in the table is [20] which is a dual-band antenna and has a gain which is lower than the proposed antenna.

**Table 2:** Comparison of proposed antenna with other antennas reported in literature.

Ref	Size (mm^3^)	Resonant Frequency(GHz)	Operational Bandwidth (GHz)	Gain (dBi)	Flexibility	Reconfigurable
[8]	30×27×1.6	2.6/4.4	2.5–10.5	4	No	No
[9]	40×37×1.6	4.5/6.5	3.5–4.7/5.2–6.75	5/4.5	No	Yes
[10]	22×22×1.6	3.5/5.5	3.1–3.8/5.2–5.75	1.5/2.2	No	No
[11]	28×24×1.6	4.5/7.5	3.75–9	4.5	No	No
[12]	40×28×0.4	2.5/6	2.1–3.1/4.4–7.7	2.2/4.5	No	No
[13]	32×25×0.254	1.88/3.1	1.8–1.95/2–2.24	-	No	No
[15]	100×100×2.5	2.45/3.5	2.3–2.5/3–4.5	5.5/5	No	Yes
[16]	27×16×1.6	2.4/5.2	2.3–2.6/5.1–5.3	3	No	Yes
[17]	30×30×1.9	2.5/3.5	1.8–4.5	3.7	No	Yes
[18]	40×40 ×0.762	5/5.5	4.9–5.1/5.4–5.55	5.6	No	Yes
[19]	40×26×1.6	2.8/4.8	2.5–3.5/4.25–5	7.68	No	No
[20]	30×40×0.79	3.5/5.5	3–4.1/3.4–7.6	3.02	Yes	Yes
[21]	53×35×1.6	2.45/3.5/5.2	2.4–2.6/3.3–3.6/5–5.3	1.7/3.4	No	Yes
[22]	42×36×2.4	2.45/3.3	2.2–2.5/3.28–3.45	4.4/4	No	Yes
[23]	40×40×1.6	2.45/5.2	2.4–2.475/5.1–5.25	2.18/0.9	No	No
[30]	80×80×1.6	1.8/2	0.5–2.43	-3.62/-1.7	No	Yes
[31]	50×50×1.6	3.5/5.5	2.9–4/5.1–6.19	4.2/3.5	No	Yes
[32]	20×15×0.254	4.2/7.1	4–7.5	-	Yes	No
[33]	17×18×0.8	3.5/5.8	3.3–3.7/5.7–5.94	1.5/0.9	No	No
[34]	37×49×1.6	5.8	3.5–5.97	1.8	No	Yes
This Work	25×30×0.064	2.45/3.3/4.1	2.1–5.2	>4	Yes	Yes

## 4. Conclusion

An electronically reconfigurable planar antenna has been successfully demonstrated for wireless applications. The triband antenna can be configured using PIN diode switches to operate in various bands including ISM, WLAN, WiMAX, and sub 6GHz 5G. This is achieved activating the two PIN diodes in one of four modes. It has been experimentally verified the flexible quality of the antenna does not undermine the performance of the antenna thus enabling it to be mounted on curved surfaces. The proposed antenna has a gain > 3.4 dBi and radiation efficiency > 94%. The antenna radiates omnidirectionally in the E-plane and bi-directionally in the H-plane.
